# Silica Nanoparticles for Intracellular Protein Delivery: a Novel Synthesis Approach Using Green Fluorescent Protein

**DOI:** 10.1186/s11671-017-2280-9

**Published:** 2017-09-25

**Authors:** Sarah Schmidt, Isabella Tavernaro, Christian Cavelius, Eva Weber, Alexander Kümper, Carmen Schmitz, Jana Fleddermann, Annette Kraegeloh

**Affiliations:** 1 Leibniz Institute for New Materials, Campus D2 2, 66123 Saarbrücken, SL Germany; 2 nanoSaar Lab GmbH, Comotorstr. 2, 66802 Überherrn, SL Germany

**Keywords:** Core-shell silica nanoparticles, Green fluorescent protein, Protein delivery, Bioimaging

## Abstract

In this study, a novel approach for preparation of green fluorescent protein (GFP)-doped silica nanoparticles with a narrow size distribution is presented. GFP was chosen as a model protein due to its autofluorescence. Protein-doped nanoparticles have a high application potential in the field of intracellular protein delivery. In addition, fluorescently labelled particles can be used for bioimaging. The size of these protein-doped nanoparticles was adjusted from 15 to 35 nm using a multistep synthesis process, comprising the particle core synthesis followed by shell regrowth steps. GFP was selectively incorporated into the silica matrix of either the core or the shell or both by a one-pot reaction. The obtained nanoparticles were characterised by determination of particle size, hydrodynamic diameter, ζ-potential, fluorescence and quantum yield. The measurements showed that the fluorescence of GFP was maintained during particle synthesis. Cellular uptake experiments demonstrated that the GFP-doped nanoparticles can be used as stable and effective fluorescent probes. The study reveals the potential of the chosen approach for incorporation of functional biological macromolecules into silica nanoparticles, which opens novel application fields like intracellular protein delivery.

**Electronic supplementary material:**

The online version of this article (10.1186/s11671-017-2280-9) contains supplementary material, which is available to authorized users.

## Background

In recent years, the encapsulation of proteins into micro- and nanoparticles has gained wide attention due to the broad application potential of such materials as biosensors [1] or bioreactors [2], and further in the fields of controlled protein delivery [3], intracellular protein delivery [4] and tissue engineering [5]. In many of these applications, the catalytic activity of encapsulated enzymes is one basic function of such materials. In contrast, pharmaceutically relevant proteins, peptide hormones or antibodies as potential cargo of such nanomaterials exert their function by specific binding of targets within tissues or cells. Therefore, one prerequisite of all of these applications is the maintenance of an intact conformation and functionality of the cargo proteins. Nanostructured systems have become one of the most rapidly developing areas in biomedical research, due to their small size, large specific surface area and other unique properties [6]. Hence, the development of new particulate carriers to improve functionality and stability of the designed systems is an important topic in the field [7]. The matrix of nanoparticulate carriers can be based on biomacromolecular or organic components like carbohydrates, lipids or polymers, forming systems like solid-lipid nanoparticles, liposomes or dendrimers. Furthermore, nanostructured systems can also be based on inorganic materials like metals or oxides [8]. All of these material systems have to fulfil various common as well as specific requirements. First of all, the matrix materials have to be biocompatible in order to facilitate safe applications [9]. Secondly, they have to be stable enough to fulfil their function as carrier materials along the life cycle of the systems. Furthermore, they have to provide the capacity for significant protein load and retention as well as for controlled protein release [10].

Besides the attachment of proteins to the surface of nanoobjects via adsorption or covalent binding [11], proteins can be entrapped within nanostructures, thereby enhancing their stability and enzymatic activity [2]. Nanoentrapment can be achieved by hydrolysis and condensation of a silica precursor via sol-gel processing [12] or via water-in-oil microemulsion approaches, causing polymerisation of the enzyme surrounding shell at the water-oil interface [13]. In these methods, the entrapment of proteins can occur by two different chemical approaches, using covalent or non-covalent binding processes [14]. In particular, amorphous silicon dioxide is a promising carrier material for proteins, due to its high biocompatibility, inertness and mechanical stability [15]. Various routes, especially biomimetic approaches for enzyme encapsulation into silicon dioxide have been followed [2, 16], whereby the release profile of the enzymes is controlled by chemical reactions of the linker or the degradation of the silica matrix. Mesoporous materials have also been used as matrix to immobilise enzymes within pores of 2–50 nm [13, 17]. Cargo release from mesoporous nanoparticles can be adjusted by using the “gatekeeper” strategy or modifying the inner surface of the pores to control the binding affinity with drugs [10b]. Nevertheless, the pore size may limit the loading of enzymes into the performed mesoporous silica scaffolds [18], which is why new strategies are recently under investigation for protein delivery.

As silica nanoparticles are widely used for bioimaging [19], the incorporation of fluorescent proteins constitute one option for the generation of biocompatible fluorescent probes. For example, the incorporation of green fluorescent protein (GFP) into silica nanoparticles via reverse emulsion techniques has been described in literature [20]. These studies indicate that the incorporation of GFP into the silica particle matrix not only enhance the fluorescence intensity of the protein but also its thermal stability, stability against chemical denaturation and protease treatment. Nevertheless, the method is less suited for the synthesis of well-defined silica nanoparticles in the lower nanoscale range with a narrow size distribution. Additionally, the synthesis conditions include contact to surfactants, alcohols or high alkaline bases as well as high temperatures that all may not be compatible with the incorporation of susceptible proteins [20, 21].

Therefore, we report on a novel approach for the preparation of protein-doped silica nanoparticles, using GFP as a model protein. For this purpose, we used a one-pot synthesis at mild synthesis conditions (room temperature, low salinity) followed by dialysis steps for purification. The approach is characterised by its potential to prepare protein-entrapped silica nanoparticles exhibiting a narrow size distribution in the size regime below 50 nm.

## Methods

### Materials

All chemicals were used as purchased from Sigma-Aldrich (Taufkirchen, Germany) and without further purification. For all synthesis and purification steps, ultrapure water (18.2 MΩ, Milli-Q water purification system type ELIX 20, Millipore Corp., USA) was used.

### Preparation of GFP

GFP was obtained by protein expression and subsequent purification as described elsewhere [22]. Briefly, GFP including a N-terminal His6-tag was expressed using a high-level bacterial expression vector based on the pQE vector system (Qiagen, Hilden, Germany) in *E. coli* XL1-Blue and purified by Ni-charged affinity chromatography (Qiagen, Hilden, Germany). Subsequently, the protein was transferred to a concentrator device (3 kDa molecular weight cut off (MWCO) membrane, Pall, Dreieich, Germany) for buffer exchange. GFP was washed three times by addition of 15 mL ʟ-arginine and sodium bicarbonate solution, respectively, and subsequently recovered in 3 mL of the ʟ-arginine/sodium bicarbonate solution. After that, the GFP-suspension was filtered into sterile tubes through sterile 0.22 μm cellulose acetate filters (Carl Roth, Karlsruhe, Germany). Prior to use, the protein concentration was adjusted to 1 mg mL^−1^ in either 7.2 mmol L^−1^ ʟ-arginine (pH = 10.3) or 10.0 mmol L^−1^ NaHCO_3_ (pH = 9.2) solution.

### Syntheses and Purification of Nanoparticles

Silica nanoparticles were prepared according to a modified protocol described previously [23]. Briefly, tetraethoxysilane (TEOS), used as nonpolar precursor, was hydrolysed in a biphasic water/cyclohexane system mediated by ʟ-arginine catalysis.

### Preparation of Core Particles

In a three-neck round-bottom flask, 91 mg (0.52 mmol) ʟ-arginine was dissolved in 69 mL of water, before 4.5 mL of cyclohexane was added as a top layer. The reaction mixture was heated to 40 °C under stirring. After addition of 5.5 mL (24.63 mmol) TEOS, the mixture was kept under these conditions for further 20 h.

### Silica Shell Layers

For subsequent shell-growth steps, either the core particles or particles resulting from the first shell-growth step were used. For shell growth, 14 mg (0.08 mmol) ʟ-arginine was dissolved in 36 mL of water and 10 mL of previously prepared particle dispersions was added. After addition of 5 mL cyclohexane, the mixture was heated to 40 °C. After addition of 3.52 mL (15.8 mmol) TEOS, the mixture was stirred for further 20 h.

**Preparation of GFP-doped nanoparticles.** For preparation of GFP-doped nanoparticles, 30 min after addition of TEOS, 200 μg (6.9 nmol) of GFP was added.

### Particle Purification

The nanoparticles were purified by subsequent dialysis against water (4 L, water exchange after 30, 90 and 180 min) for 4 h using a cellulose hydrate membrane (Nadir-dialysis tubing, MWCO 10 kDa, Carl Roth, Karlsruhe, Germany). Finally, the nanoparticles were filtered into sterile flasks using sterile 0.22 µm cellulose acetate membrane filters (Carl Roth, Karlsruhe, Germany).

### Transmission Electron Microscopy (TEM)

Morphology and mean particle diameter were determined using a JEM-2100F microscope (JEOL, Freising, Germany). The particle size distribution was determined on a random sample of 50 nanoparticles using the X-ImageJ software (Version: 1.45 s, winPenPack X-ImageJ Launcher from the National Institute of Health (http://rsb.info.nih.gov/ij/).

### Hydrodynamic diameter

The hydrodynamic diameter of the nanoparticles was recorded using a Zetasizer Nano ZSP (Malvern Instruments, Herrenberg, Germany). Prior to measurements, particle dispersions were diluted 1:10 in water. Measurements were performed at 25 °C. Each sample was measured 3 × 15 times. The diameter was determined by calculation of the volume distribution. This was converted from the intensity size distribution using Mie theory.

### ζ-potential

The ζ-potential was measured using the same instrument with the above-described conditions, except that the samples were diluted in 0.01 M KCl (9:1).

### Analytical Ultracentrifugation (AUC)

To measure the sedimentation velocity, a modified Beckman-Coulter XL-80 K with aAnTi60 rotor. For the experiments, the temperature was set on 20 °C, the velocity was set on 10,000 rpm and 21 scans were done. The wavelengths were set on 261 nm for silica and 488 nm for GFP detection.

### Fluorescence Spectroscopy

Fluorescence spectra of nanoparticles, pure GFP and filtrates from leaching experiments were recorded using the Fluoromax-3 spectrofluorometer (Spex, Horiba Scientific, Oberursel, Germany). For measurements, pure GFP, particle dispersions and filtrate were diluted 1:10 in water. The excitation wavelength was set to 488 nm, and the spectrum was recorded in a spectral range of 498 to 800 nm.

### Fluorescence Quantum Yields

Quantum yields of the obtained nanoparticles and pure GFP were determined using the relative method of Williamson et al. [24]. As reference for GFP, rhodamine 6G and Atto488 were used. Comparative measurements were taken using non-doped nanoparticles that have been mixed with the reference dye. Fluorescence spectra were recorded using an excitation wavelength of 450 nm. Additional UV/vis measurements were taken using a Varian Cary 300 Scan UV (Agilent Technologies, Darmstadt, Germany).

For calculation of the quantum yield, Eq. 2 was used.

2 $$ {\varPhi}_P={\varPhi}_S\bullet \frac{{\mathrm{slope}}_S}{{\mathrm{slope}}_P}\bullet {\left(\frac{n_P}{n_S}\right)}^2 $$

Here, *φ*_P_ is the quantum yield of the product, *φ*_S_ the quantum yield of the reference. The terms slope_S_ and slope_P_ represent the slopes derived from the plots of integrated fluorescence intensity vs. absorbance of reference and product, respectively. *n*_P_ and *n*_S_ correspond to the refractive index of the used solvent [25].

### Protein Leakage

For leaching experiments, undiluted particle dispersions were ultrafiltered through modified polyether sulphone membranes (MWCO = 100 kDa or 300 kDa, Pall, Dreieich, Germany) by centrifugation (16,000 g, 5 min).

### Thermal Stability

For analysis of thermal stability, nanoparticles and pure GFP were kept for 0 and 24 h at either 20 or 60 °C. Nanoparticles and pure GFP were diluted as described above.

### Photobleaching

To investigate the stability of GFP-doped nanoparticles and pure GFP against photobleaching, the solutions were exposed to light emitted from seven green LEDs over a period of time up to 20 min. The fluorescence intensity of samples taken at *t* = 0, 2 and 20 min was measured.

### Stability Against Protein Degradation

To determine the stability of GFP against proteinase K the pure GFP, unlabelled silica nanoparticles (C_U_S_1U_S_2U_) with additional GFP and three times labelled silica nanoparticles (C_F_S_1F_S_2F_) were used in the same GFP concentrations and same amount of particles. All samples were diluted 1:100. For the amount of 10 GFP molecules, one proteinase K molecule was chosen. Before the addition of the enzyme, one sample was measured with the above conditions. After addition, the measurements were done after *t* = 0, 15, 30, 45, 60 and 90 min.

### Cellular Uptake Experiments

To determine internalisation of nanoparticles and GFP by cells, cellular uptake experiments were carried out using the lung carcinoma cell line A549 (ACC-107).

### Cultivation of Cells

A549-cells (DSMZ, Braunschweig, Germany) were cultured in T75-flasks (Greiner bio-one, Frickenhausen, Germany) using Dulbecco’s Modified Eagle Medium (DMEM, Thermo-Fisher-Scientific, Waltham, MA, USA) containing 10% foetal calf serum (FCS). 2 × 10^4^ cm^−2^ A549-cells were seeded on cover slips in 12-well plates and were cultured for 24 h. Cells were then treated with GFP-doped nanoparticles and GFP solution in 1 mL medium for 24 h. The SiO_2_ concentration of the nanoparticles was 37 μg mL^−1^ while the GFP concentration was 5 μg mL^−1^ for both nanoparticles and pure GFP. After treatment, cells were washed twice with phosphate buffered saline (PBS).

### Sample Preparation and Confocal Imaging

Cells were fixed with 4% paraformaldehyde in PBS for 20 min at room temperature. For staining of the cell membrane, tetramethylrhodamine-conjugated WGA (wheat germ agglutinin (2 μg mL^−1^ (in PBS), W849, Thermo-Fisher-Scientific (Invitrogen), Waltham, MA, USA) was added and incubated for 10 min at room temperature. After three washing steps with PBS, cells were washed triply with PBS and mounted on glass slides with Mowiol/DABCO (Carl Roth, Karlsruhe, Germany).

Confocal images were taken on a TCS SP5 system (Leica, Wetzlar, Germany). For imaging, a 63× oil-immersion-objective (*n* = 1.518) was used. Sequential scans were taken using the argon-ion laser line at *λ* = 488 nm (25%) for excitation of GFP, and a diode-pumped solid state laser at *λ* = 561 nm (25%) for excitation of tetramethylrhodamine.

## Results and Discussion

**Scheme 1:**
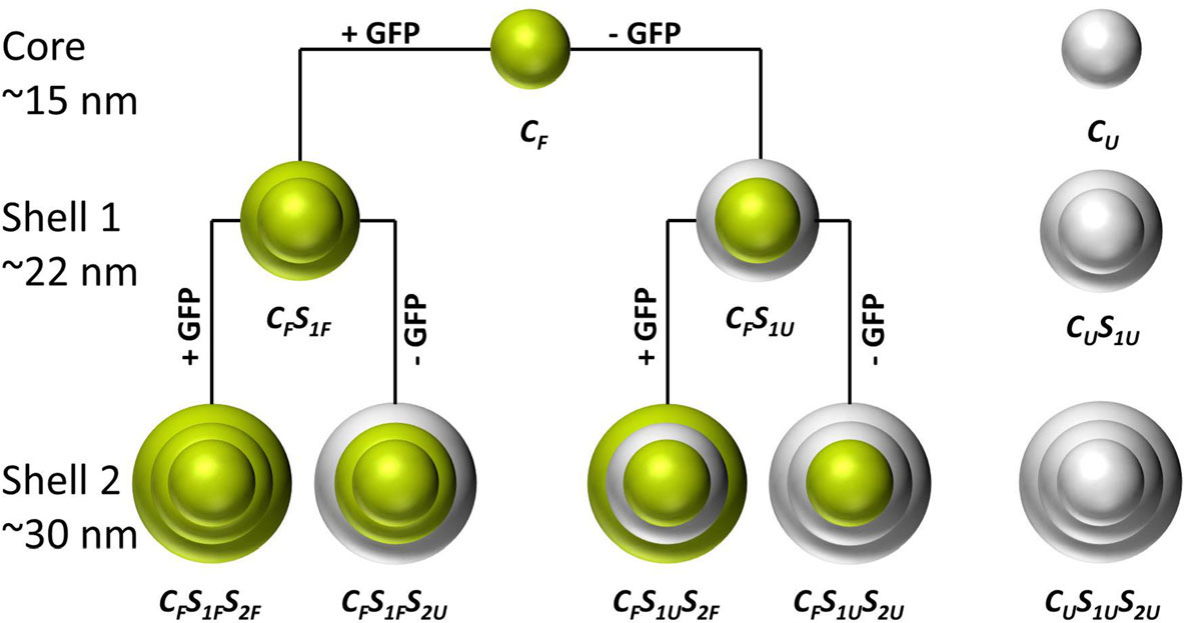
Overview of the synthesised particles and their particle structure. Green colour indicates embedding of GFP into either the core or the shells, respectively. Grey colour is representing the shells without any GFP (C_F_ = core fluorescent, C_U_ = core unlabelled, S_F_ = shell fluorescent, S_U_ = shell unlabelled, S_1_ = first shell layer, S_2_ = second shell layer)

This study aims at functionalising silica nanoparticles with GFP under suitable conditions that maintain the biochemical characteristics and functionality of the protein. In previous work, we synthesised near IR dye-doped monodisperse fluorescent silica nanoparticles in the size range between 15 and 80 nm, using a ʟ-arginine controlled hydrolysis of tetraethoxysilane (TEOS) in a biphasic cyclohexane/water system [26]. Here, we have adopted this synthesis procedure to embed GFP, as a model protein, into the silica matrix. In Scheme 1, the procedure for the particle synthesis is depicted schematically. GFP-doped and non-doped structures (core/shells) are highlighted in green and grey, respectively. In a first step, GFP-doped silica core particles (C_F_) were obtained. Subsequent regrowth steps (C_F_S_1_ and C_F_S_1_S_2_) allowed the synthesis of larger particle sizes. During the first regrowth step, the shell was modified either with (C_F_S_1F_) or without (C_F_S_1U_) incorporation of protein. Similarly, in the second regrowth step, either a labelled (C_F_S_1F_S_2F_, C_F_S_1U_S_2F_) or an unlabelled (C_F_S_1F_S_2U_, C_F_S_1U_S_2U_) shell was added. These variations allow an excellent control over the amount of embedded protein and its tailored arrangement into designated shells or the particle core. Furthermore, pure silica nanoparticles without any embedded GFP (C_U_, C_U_S_1U_, and C_U_S_1U_S_2U_) were synthesised to investigate a potential influence of the protein embedding on the particle properties. In addition, for all these steps, the GFP was dissolved in two different buffer systems (ʟ-arginine and NaHCO_3_) of various pH values, to determine the influence of the protein solvent on the particle syntheses, the morphology, the fluorescence intensity, the emission wavelength and the ζ-potential.

### Nanoparticle Characterisation

#### Determination of Physical Particle Attributes

**Fig. 1:**
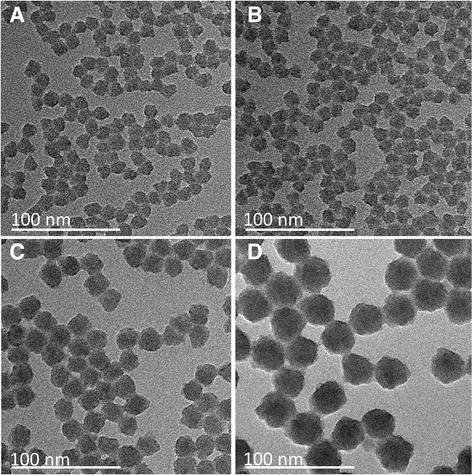
TEM images of three generations of GFP-ʟ-arginine modified nanoparticles and core particles of GFP(NaHCO_3_) modified nanoparticles. In **a**, **c** and **d**, the three generations of GFP(ʟ-arginine) are shown: C_F_ core particles (**a**, d_TEM_ = 15.5 ± 1.1 nm); C_F_S_1F_ nanoparticles after the first regrowth step (core + shell 1) (**c**, d_TEM_ = 23.5 ± 2.0 nm) and C_F_S_1F_S_2F_ after the second regrowth step (core + shell 1 + shell 2) (**d**, d_TEM_ = 35.3 ± 2.0 nm). In **b**, the GFP(NaHCO_3_)-labelled core nanoparticles (d_TEM_ = 15.2 ± 1.2 nm) are shown

To describe the particle size and morphology after incorporation of GFP and in order to determine the influence of the two different buffer systems on these properties, TEM images were recorded (Fig. 1). Further TEM images of GFP(NaHCO_3_) modified, GFP(ʟ-arginine) modified, and unlabelled nanoparticles are presented in the SI (Additional file 1: Figure S1, Additional file 2: Figure S2, Additional file 3: Figure S3, Additional file 4: Figure S4). Following the synthesis procedure with two regrowth steps, three different particle sizes were obtained. The core particles had a size of about 15 nm, the particles after the first regrowth step about 22 nm and the particles after the second step about 32 nm. In summary, all nanoparticles were approximately spherical and exhibited a narrow size distribution (*p* < 10%). The three generations of fully dyed GFP(ʟ-arginine) nanoparticles (C_F_, C_F_S_1F_, and C_F_S_1F_S_2F_) and the GFP(NaHCO_3_) (C_F_) core nanoparticles were chosen as model.

Comparing the sizes of the different GFP-doped and unlabelled nanoparticles (Table 1), it is noteworthy that the same number of regrowth steps resulted in the same mean particle size, independently of the presence of protein or the used buffer solution. The unlabelled particles had also similar sizes (C_U_: d_TEM_ = 13.4 ± 0.4 nm, d_DLS_ = 10 ± 3 nm; C_U_S_1U_: d_TEM_ = 20.9 ± 1.3 nm, d_DLS_ = 20 ± 6 nm; C_U_S_1U_S_2U_: d_TEM_ = 33.2 ± 1.0 nm, d_DLS_ = 38 ± 10 nm).

**Table 1:** Diameters [nm] of the various particle types derived from TEM and DLS measurements

	GFP(ʟ-arginine)		GFP(NaHCO_3_)	
	Diameter [nm]		Diameter [nm]	
	TEM	DLS	TEM	DLS
C_F_	15.5 ± 1.1	10 ± 3	15.2 ± 1.2	11 ± 4
C_F_S_1F_	23.5 ± 2.0	22 ± 7	22.7 ± 2.1	21 ± 6
C_F_S_1U_	21.1 ± 1.6	19 ± 5	21.9 ± 1.7	20 ± 6
C_F_S_1F_S_2F_	35.3 ± 2.0	35 ± 9	33.1 ± 1.8	33 ± 9
C_F_S_1F_S_2U_	31.7 ± 1.6	35 ± 9	31.4 ± 1.3	33 ± 9
C_F_S_1U_S_2F_	34.1 ± 1.6	38 ± 10	32.7 ± 1.3	37 ± 9
C_F_S_1U_S_2U_	33.1 ± 1.7	37 ± 9	32.1 ± 0.7	37 ± 9

Mean values ± standard deviation are given

In conclusion, it was demonstrated that the incorporation of protein into the silica matrix and the buffer solution, in which the protein was provided, had no significant influence on the resulting particle size and morphology.

To the best of our knowledge, there are no other GFP-embedded silica nanoparticles described in literature, exhibiting similar small sizes as well as equally narrow size distributions (< 10%) [20, 27]. Such small nanoparticles bear a promising application potential in the field of intracellular protein delivery as well as in cancer diagnosis and therapy [28].

### ζ-Potential

**Fig. 2:**
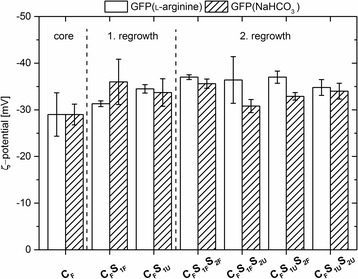
ζ-potential [mV] of labelled nanoparticles. The nanoparticles were prepared starting from GFP dissolved in either 7.2 mM ʟ-arginine or 10 mM NaHCO_3_. *Error bars* indicate the standard deviation derived from three measurements

The ζ-potential of all nanoparticles was determined through calculations using their electrophoretic mobility. All types of doped nanoparticles exhibited a negative ζ-potential with absolute values ranging from − 28 to − 36 mV (Fig. 2). In comparison, the ζ-potential of unlabelled particles indicate very similar values with − 35.5 ± 2.0 mV for the core particles, − 34.0 ± 3.7 mV after the first regrowth step and − 34.5 ± 1.2 mV after the second regrowth step. These highly negative ζ-potential (< − 28 mV) values indicate a high stability of the nanoparticles against agglomeration due to electrostatic repulsion. Compared to the ζ-potential of unlabelled nanoparticles, the data indicates that neither the resulting particle size nor the incorporation of GFP into the particle matrix of either particle core or shell had a significant influence on the particle charge.

### Spectroscopy Studies

#### Fluorescence Spectroscopy

**Fig. 3:**
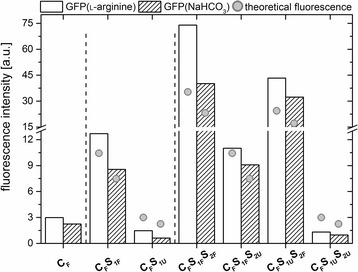
Normalised fluorescence intensity of the emission maximum at 508 nm, for each of the various particle systems. Further, the theoretical fluorescence intensity (*grey points*) of the particles in relation to the increase of particle volume is shown

All GFP-doped silica nanoparticles exhibited a similar emission maximum (*λ*_em_ = 508 nm), which was also comparable to the emission maximum of free GFP (SI, Additional file 5: Figure S5). To compare the fluorescence intensity of the various labelled nanoparticles, the nanoparticle concentration was normalised (calculations in SI 5.). As expected, the stepwise addition of labelled shells caused an increase in the fluorescence of the nanoparticles (Fig. 3).

Nanoparticles with a labelled core only, but with non-doped shells, exhibited the lowest fluorescence. Nanoparticles with one additional, labelled shell exhibited an intermediate fluorescence, and nanoparticles with two labelled shells showed the strongest fluorescence (Fig. 3). Remarkably, the addition of an outer non-doped shell seemed to slightly reduce the fluorescence of nanoparticles as compared to nanoparticles possessing a doped outer layer. This effect might be elicited by shielding effects of the unlabelled silica shell. In summary, the addition of GFP-doped shells to core particles caused an increase in fluorescence intensity of the resulting nanoparticles that seemed to be correlated with the volume change accompanying nanoparticle growth.

The embedding of GFP initially dissolved in ʟ-arginine after purification resulted in a 1.3-fold higher fluorescence intensity of the resulting nanoparticles as compared to the nanoparticles obtained via the analogue embedding process starting from GFP dissolved in NaHCO_3_. Similarly, GFP diluted in ʟ-arginine exhibited a higher fluorescence intensity compared to GFP diluted in NaHCO_3_ (Additional file 5: Figure S5). The effect might be explained by the different pH values of the buffers (pH_ʟ-arginine_ = 10.3, pH$_{{\mathrm{NaHCO}}_3}$ = 9.2).

For this reason, the fluorescence of the pure GFP was systematically measured as a function of the pH value (SI, Additional file 6: Figure S6). The data showed a hyperbolically shaped increase of the fluorescence with increasing pH in the range of pH 5.5 - 10.5. The results are consistent with other reports on pH dependent fluorescence of GFP. For wild-type GFP, it has been reported that the fluorescence is unaltered in the range of pH 6 - 10 but decreases at lower pH and increases at pH values > 10 [29]. In addition, the pH sensitivity of GFP could be modified by introduction of point mutations [30]. The GFP used in this study possesses three-point mutations as compared to the *Aequorea* wild-type protein, namely S2A, F64L, S65T. Of these, the substitution of serine at position 65 against threonine has been shown to increase the fluorescence intensity of the protein, when excited at 480 nm, as this amino acid is involved in formation of the chromophore. In addition, the S65T/F64L variant exhibits a pH-dependent fluorescence [30]. The GFP-doped nanoparticles (C_F_) exhibited a comparable pH-dependent fluorescence (Fig. 3), indicating that the mechanism of pH dependence was unaffected by the embedding process.

#### Fluorescence Quantum Yield

**Fig. 4:**
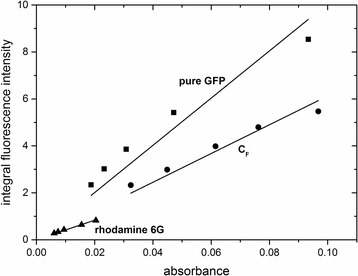
Plots of integrated fluorescence intensity of GFP-doped particles and pure GFP versus absorbance at 488 nm. Rhodamine 6G was used as a reference. The linear correlation was fitted by the *straight lines*. The corresponding linear equations are as follows: *y*_pureGFP_ = 1.00554 × 10^10^ × *x*, *R* ^2^ = 0.97712; y$_{C_F{S}_{1F}}$ = 6.12332 × 10^9^ × *x*, *R* ^2^ = 0.99331; *y*_rhodamin6G_ = 4.1772 × 10^9^ × *x*, *R* ^2^ = 0.99678

In order to further characterise the properties of the fluorescent nanoparticles, their quantum yields were determined. This was achieved by plotting the integrated fluorescence intensity versus the absorbance at 488 nm (Fig. 4). Subsequently, the quantum yields were calculated using Eq. 2. Using rhodamine 6G as a reference, the quantum yields of the GFP-doped nanoparticles C_F_S_1F_ and the pure GFP were determined to be φ$_{{\mathrm{C}}_{\mathrm{F}}{\mathrm{S}}_{1\mathrm{F}}}$ = 0.62 and φ_pureGFP_ = 0.38, respectively. The results were confirmed by using Atto488 as a second reference (SI, Additional file 7: Figure S7). The higher quantum yield of GFP-doped nanoparticles as compared to the pure GFP seemed to be caused by the encapsulation of GFP into the silica matrix and could either be linked to the spatial immobilisation of the protein or the altered chemical environment provided by the silica matrix.

### Particle Stability

#### Protein Leakage

Leaching experiments were performed to prove the binding stability of the GFP-doped nanoparticles. After ultrafiltration through membranes with a MWCO that allows for the passing of GFP (MW~27 kDa) but retains nanoparticles, no fluorescence could be detected in the filtrate, indicating a permanent coupling of GFP to the silica matrix.

#### Analytical Ultracentrifugation

To support the obtained results and to determine the type of GFP bonding to the particle matrix, analytical ultracentrifugation was conducted. For this purpose, labelled C_F_S_1F_S_2F_ particles and unlabelled C_U_S_1U_S_2U_ particles mixed with GFP were measured at the same particle and GFP concentrations. The results (Additional file 8: Figure S8 in the SI) indicate that most of the GFP molecules are embedded into the silica matrix during the synthesis.

#### Thermal Stability

**Fig. 5:**
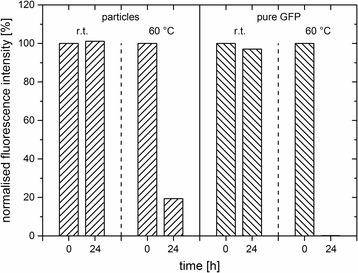
Influence of temperature (r.t., 60 °C) on the fluorescence of GFP-doped particles (C_F_, ʟ-arginine) and pure GFP. The normalised fluorescence intensity [%] of the emission maximum at 508 nm versus time [h] is shown

To determine their thermal stability, the fluorescence signals of C_F_ in comparison to pure GFP were measured after incubation at room temperature and 60 °C respectively (Fig. 5). After 24 h at room temperature, no decrease in the fluorescence of both samples was detectable, indicating no influence on the protein stability. However, after 24 h at elevated temperature of 60 °C, only 20% of the initial fluorescence intensity of C_F_ could be observed, whereas no fluorescence signal of pure GFP reverted. This strongly indicates a higher thermal stability of GFP-embedded silica compared to pure GFP. Since an elevated temperature leads to a significant increase in the thermal motions of the protein molecule, which can disrupt its structure, it is hypothesised that the surrounding silica matrix protected the GFP against external influences by spatial constraints.

#### Photostability

**Fig. 6:**
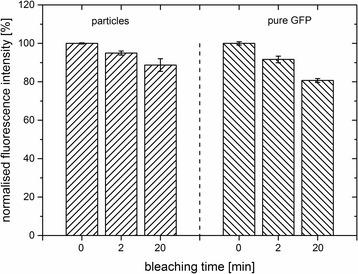
Photostability of GFP-doped nanoparticles (C_F_) and pure GFP in ʟ-arginine. The normalised fluorescence intensity [%] of the emission maximum at 508 nm was measured after exposure to LED light for the given times. Data are mean values. *Error bars* indicate the standard deviation

Furthermore, the photostability of the samples was tested. For measurements, the nanoparticle stock suspension (C_F_, ʟ-arginine) was diluted tenfold. Pure GFP was diluted in ʟ-arginine according to the calculated concentration of GFP in the nanoparticle suspension. After exposure of the samples to light of a green LED array over a period of time up to 20 min, the fluorescence intensity was measured (Fig. 6). Within 20 min, the fluorescence intensity of the nanoparticle suspension decreased only slightly. After 20 min, 89% of the initial fluorescence (100%) of the nanoparticles was preserved. In comparison, the pure GFP seemed to be more affected by light exposure. After 20 min, only 81% of the initial fluorescence of pure GFP remained. This result indicated, that GFP, when embedded into silica nanoparticles, was better protected from photochemical alterations induced by the LED light than the pure protein.

#### Stability Against Protein Degradation

**Fig. 7:**
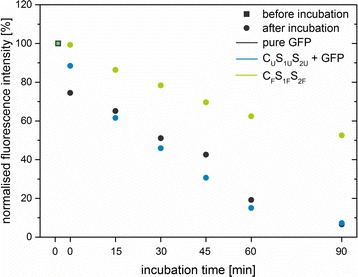
Stability against protein degradation of pure GFP (*grey*), unlabelled particles mixed with GFP (C_U_S_1U_S_2U_, *blue*), and GFP-doped silica nanoparticles (C_F_S_1F_S_2F_, *green*). The normalised fluorescence intensity [%] of the emission maximum at 508 nm was plotted against the incubation time [min] with proteinase K

As a further characterisation step, the degradation of GFP in the presence of proteinase K was tested. Therefore, three different systems were used (pure GFP, unlabelled C_U_S_1U_S_2U_ mixed with GFP and labelled C_F_S_1F_S_2F_). For all systems, equal amounts of GFP and particles were used. After 90 min of incubation, the fluorescence intensity of pure GFP and unlabelled particles with added GFP decreased to 5 - 7% of the initial fluorescence intensity, whereas the one of the labelled particles decreased to 52% (Fig. 7). This result indicates that the GFP is protected by the silica matrix and is degraded slower than free GFP in presence of proteolytic enzymes.

To conclude, the encapsulation of GFP into silica matrix appeared to bring about significant advantages: The stability of the protein was increased not only against elevated temperatures and light-induced photobleaching but also against the degradation through enzymes. Therefore, the silica matrix seems to protect the embedded GFP as compared to the free GFP.

### Cellular Uptake Experiments

**Fig. 8:**
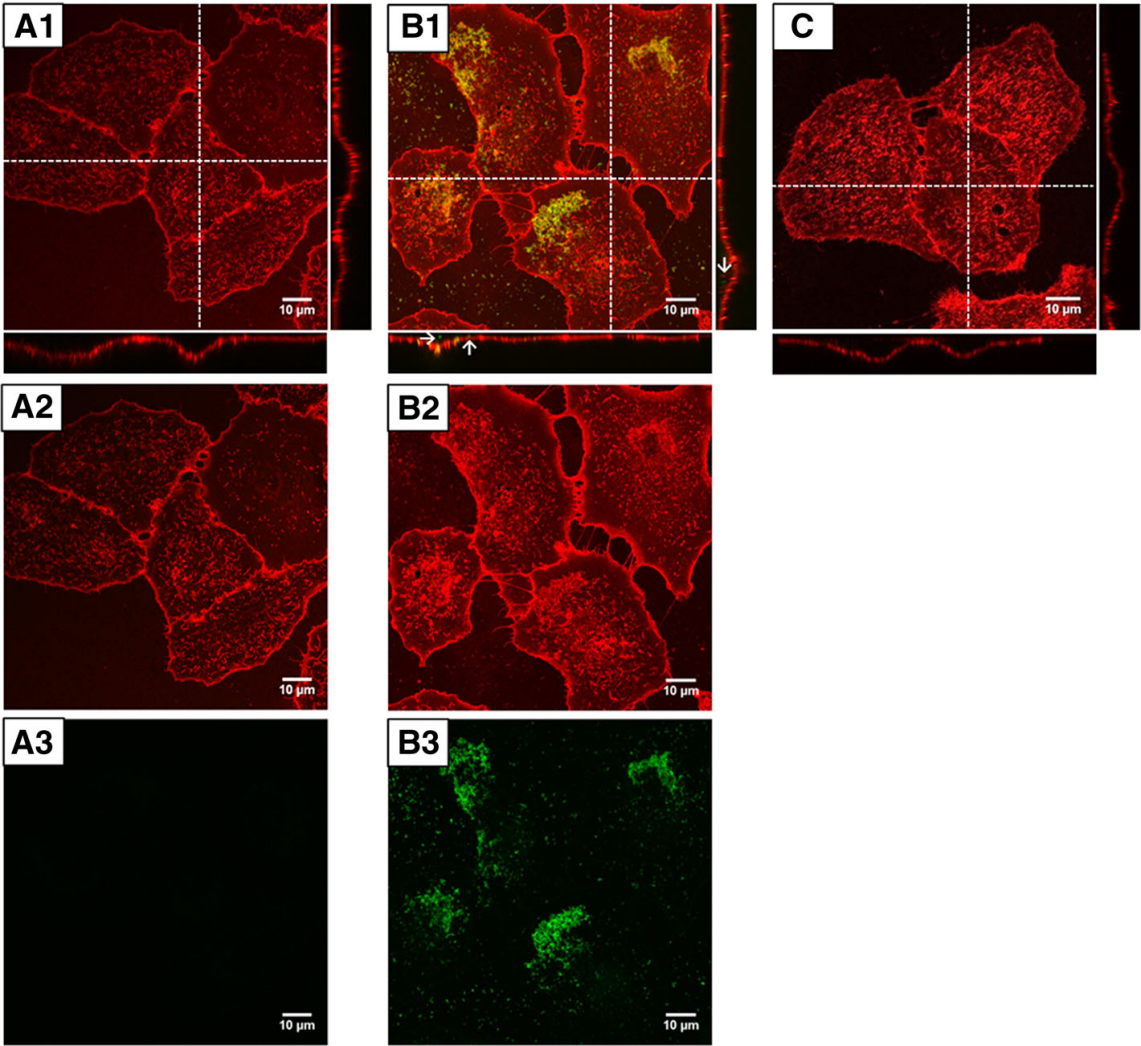
Confocal microscopy images of A549 cells after 24 h exposure to GFP dissolved in ʟ-arginine (**A1**–**A3**) and GFP-doped nanoparticles C_F_S_1F_S_2F_ (**B1**–**B3**), and control cells (**C**). *Top* (1): merge-images; *middle* (2): Cell membrane (WGA): *red*; *bottom* (3): GFP, *green*. *Arrows* indicate internalised nanoparticles. Contrast and brightness were enhanced by using the ImageJ software

In order to determine, if the GFP-doped nanoparticles are able to deliver their cargo into cells, uptake experiments were performed (Fig. 8). A549 cells were exposed to GFP-doped nanoparticles and for comparison to the pure protein. In order to optimise the GFP load of the particles for imaging, a higher amount of GFP as compared to the nanoparticles described before was embedded into the particles. More specifically, a 20-fold amount of GFP in ʟ-arginine was used to label the second shell of the C_F_S_1F_S_2F_ particles. These nanoparticles were diluted to a final concentration of 37 μg SiO_2_ per millilitre in cell culture medium and incubated for 24 h with the cells. The amount of GFP in both samples (nanoparticles and pure GFP) was 5 μg mL^−1^.

In order to visualise the cells, the cell membrane was labelled, using tetramethylrhodamine-coupled WGA (wheat germ agglutinin). Confocal imaging was used to localise the GFP-doped nanoparticles and the pure GFP in the cells. After exposure of cells to GFP, no signal related to GFP was observed inside the cell bodies (Fig. 8a). Compared to the control cells, no difference in signal intensity of both channels could be observed (Fig. 8c).

In contrast, after exposure of the cells to the GFP-loaded nanoparticles, bright fluorescence signals were detected in the perinuclear region, indicating internalisation of the loaded nanoparticles through endocytosis. The GFP-loaded nanoparticles appeared to be excluded from the nuclear compartment. A second fraction of agglomerated nanoparticles was detected on top of the cell membrane (Fig. 8b).

In conclusion, the GFP-doped nanoparticles are internalised by the cells and are able to transport their cargo into the cells. After exposure of the cells to GFP, fluorescence signals were not detected inside the cell body. This finding is in line with the results of Pesce et al. [31], who did not observe cell-associated fluorescence after incubation of A549 cells with GFP for 24 h. The lack of cell-associated GFP signals might be due to the fact that GFP is not internalised by the cells. Alternatively, GFP fluorescence might be quenched by the low pH value present in endocytic vesicles or lysosomes or degraded by proteolytic enzymes. Therefore, the fluorescence signals of the nanoparticles might indicate a protective effect of the silica nanoparticle matrix against lysosomal degradation.

## Conclusions

In this study, a novel approach is presented for synthesis of monodisperse GFP-doped silica nanoparticles with a mean particle-core size of 15 nm. By subsequent growth steps, the particle size and the amount of embedded GFP can be varied. At the end of this procedure, the fluorescence properties of GFP are kept. Incorporation of GFP into additional outer shells results in an increase in the nanoparticle fluorescence. Coverage of the nanoparticles by non-doped shells seems to slightly decrease their fluorescence. These properties indicate the potential to incorporate cargo molecules into specific particle shells. The GFP-doped nanoparticles exhibit a higher quantum yield as compared to the pure GFP. The incorporation into the silica matrix appeared to be durable, as no leaching of protein was detected by ultrafiltration. The silica matrix also seems to improve the thermal properties and photostability of the protein. Furthermore, it is possible to encapsulate different proteins in the different shells, in order to prepare multifunctional nano-carriers. Finally, the nanoparticles are applicable for intracellular delivery of their cargo. The incorporation of proteins into the particle matrix seems to increase delivery and reduce lysosomal degradation of the cargo. Therefore, the protein-doped silica nanoparticles constitute a promising novel tool for biomedical applications of nanoparticles, especially in the field of intracellular delivery of macromolecules.

## Additional Files

Additional file 1: Figure S1. TEM images of GFP(NaHCO_3_) modified particles; nanoparticles after the first regrowth step (core + shell) with a labelled shell (C_F_S_1F_, d_TEM_ = 22.7 ± 2.1 nm) with an unlabelled shell (C_F_S_1U_, d_TEM_ = 21.9 ± 1.7 nm). (JPEG 53 kb)

Additional file 2: Figure S2. TEM images of GFP(NaHCO_3_) modified particles after the second regrowth step (core + shell + shell): C_F_S_1F_S_2F_ (d_TEM_ = 33.1 ± 1.8 nm); C_F_S_1F_S_2U_ (d_TEM_ = 31.4 ± 1.3 nm); C_F_S_1U_S_2F_ (d_TEM_ = 32.7 ± 1.3 nm) and C_F_S_1U_S_2U_ (d_TEM_ = 32.1 ± 0.7 nm). (JPEG 142 kb)

Additional file 3: Figure S3. TEM images of GFP(ʟ-arginine)-doped particles; particles after the first regrowth step (core + shell) with an unlabelled shell (C_F_S_1U_, d_TEM_ = 21.1 ± 1.6 nm) and particles after the second regrowth step (core + shell + shell) [C_F_S_1F_S_2U_ (d_TEM_ = 31.7 ± 1.6 nm), C_F_S_1U_S_2F_ (d_TEM_ = 34.1 ± 1.6 nm), C_F_S_1U_S_2U_ (d_TEM_ = 33.1 ± 1.7 nm)]. (JPEG 140 kb)

Additional file 4: Figure S4.TEM images of unlabelled particles; C_U_ $\widehat{=}$ core particles (d_TEM_ = 13.4 ± 0.4 nm), C_U_S_1U_ $\widehat{=}$ particles after the first regrowth step (core + shell) (d_TEM_ = 20.9 ± 1.3 nm) and C_U_S_1U_S_2U_ $\widehat{=}$ after the second regrowth step (core + shell + shell) (d_TEM_ = 33.2 ± 1.0 nm). (JPEG 95 kb)

Additional file 5: Figure S5. Fluorescence spectra of pure GFP at a concentration of 1 μg mL^−1^, dissolved in either ʟ-arginine at pH = 10.3 or NaHCO_3_ at pH = 9.2. (TIFF 226 kb)

Additional file 6: Figure S6. Normalised fluorescence intensity of pure GFP and GFP-doped particles (C_F_) as a function of the pH value. Excitation wavelength λ_ex_ = 488 nm, Emission wavelength λ_em_ = 508 nm. Samples were diluted in a ʟ-arginine solution. The pH value was adjusted by addition of acetic acid (33% *v*/v). (TIFF 214 kb)

Additional file 7: Figure S7.Plots of integrated fluorescence intensity of GFP-doped particles and pure GFP versus absorbance at 488 nm. Atto488 was used as a reference. Fitting of the linear correlation is indicated by the straight lines. The corresponding linear equations are: y_pureGFP_ = 1.00554 ∙ 10^10^ ∙ x, R^2^ = 0.97712; ${y}_{C_F{S}_{1F}}$ = 6.12332 ∙ 10^9^ ∙ x, R^2^ = 0.99331; y_Atto488 =_ 4.21548 ∙ 10^9^ ∙ x, R^2^ = 0.99712). (TIFF 213 kb)

Additional file 8: Figure S8. AUC spectra of the GFP modified silica nanoparticles (C_F_S_1F_S_2F_) and unlabelled silica nanoparticles mixed with GFP (C_U_S_1U_S_2U_). Same amounts of particles and GFP were used. In all spectra, the absorption was plotted against the radial position (cm). To detect the sedimentation velocity of silica nanoparticles, a wavelength of 261 nm was chosen, whereas a wavelength of 488 nm was used for the GFP detection. In **A** and **B** the sedimentation of GFP-labelled particles is shown and in **C** and **D** the sedimentation of unlabelled silica nanoparticles with additional added GFP. (TIFF 8201 kb)

## Abbreviations

AUC: Analytical ultracentrifuge
DLS: Dynamic light scattering
FCS: Foetal calf serum
GFP: Green fluorescent protein
MW: Molecular weight
MWCO: Molecular weight cut off membrane
PBS: Phosphate buffered saline
r.t.: Room temperature
TEM: Transmission electron microscopy
TEOS: Tetraethoxysilane
