# Supplementary figures and images for: Silica Nanoparticles for Intracellular Protein Delivery: a Novel Synthesis Approach Using Green Fluorescent Protein
Source: Nanoscale Res Lett. 2017 Sep 25;12:545. doi: 10.1186/s11671-017-2280-9 (PMC5612907; doi:10.1186/s11671-017-2280-9)

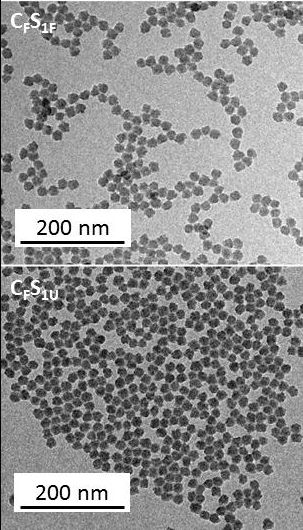

Supplement: Supplementary file 1 — TEM images of GFP(NaHCO3) modified particles; nanoparticles after the first regrowth step (core + shell) with a labelled shell (CFS1F, dTEM = 22.7 ± 2.1 nm) with an unlabelled shell (CFS1U, dTEM = 21.9 ± 1.7 nm). (JPEG 53 kb) [file 11671_2017_2280_MOESM1_ESM.jpg]

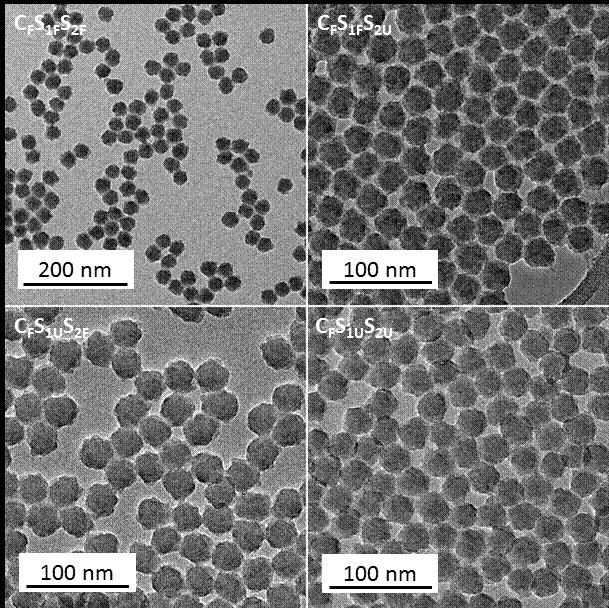

Supplement: Supplementary file 2 — TEM images of GFP(NaHCO3) modified particles after the second regrowth step (core + shell + shell): CFS1FS2F (dTEM = 33.1 ± 1.8 nm); CFS1FS2U (dTEM = 31.4 ± 1.3 nm); CFS1US2F (dTEM = 32.7 ± 1.3 nm) and CFS1US2U (dTEM = 32.1 ± 0.7 nm). (JPEG 142 kb) [file 11671_2017_2280_MOESM2_ESM.jpg]

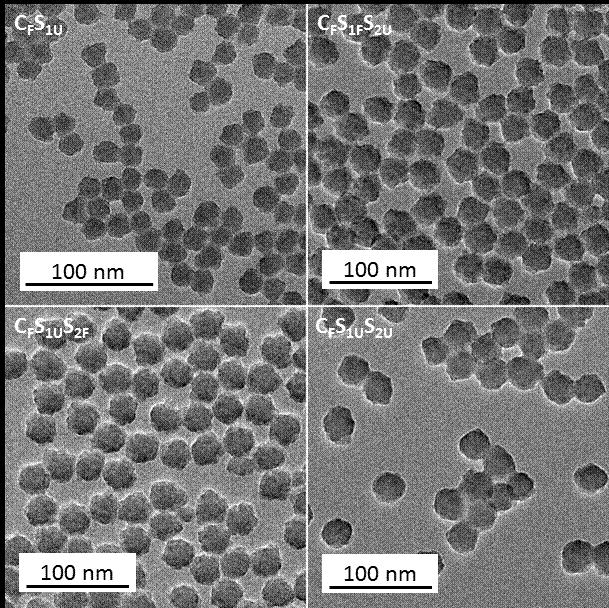

Supplement: Supplementary file 3 — TEM images of GFP(ʟ-arginine)-doped particles; particles after the first regrowth step (core + shell) with an unlabelled shell (CFS1U, dTEM = 21.1 ± 1.6 nm) and particles after the second regrowth step (core + shell + shell) [CFS1FS2U (dTEM = 31.7 ± 1.6 nm), CFS1US2F (dTEM = 34.1 ± 1.6 nm), CFS1US2U (dTEM = 33.1 ± 1.7 nm)]. (JPEG 140 kb) [file 11671_2017_2280_MOESM3_ESM.jpg]

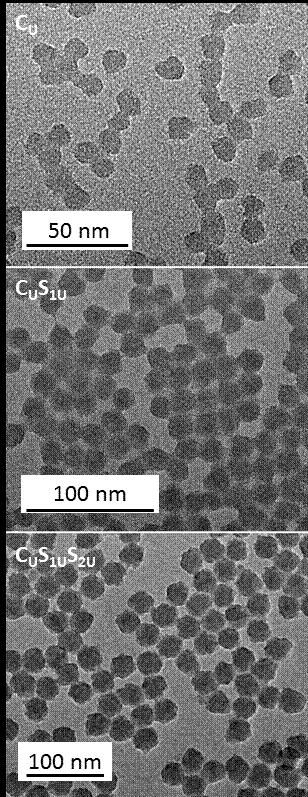

Supplement: Supplementary file 4 — TEM images of unlabelled particles; CU $\widehat{=}$=^ core particles (dTEM = 13.4 ± 0.4 nm), CUS1U $\widehat{=}$=^ particles after the first regrowth step (core + shell) (dTEM = 20.9 ± 1.3 nm) and CUS1US2U $\widehat{=}$=^ after the second regrowth step (core + shell + shell) (dTEM = 33.2 ± 1.0 nm). (JPEG 95 kb) [file 11671_2017_2280_MOESM4_ESM.jpg]

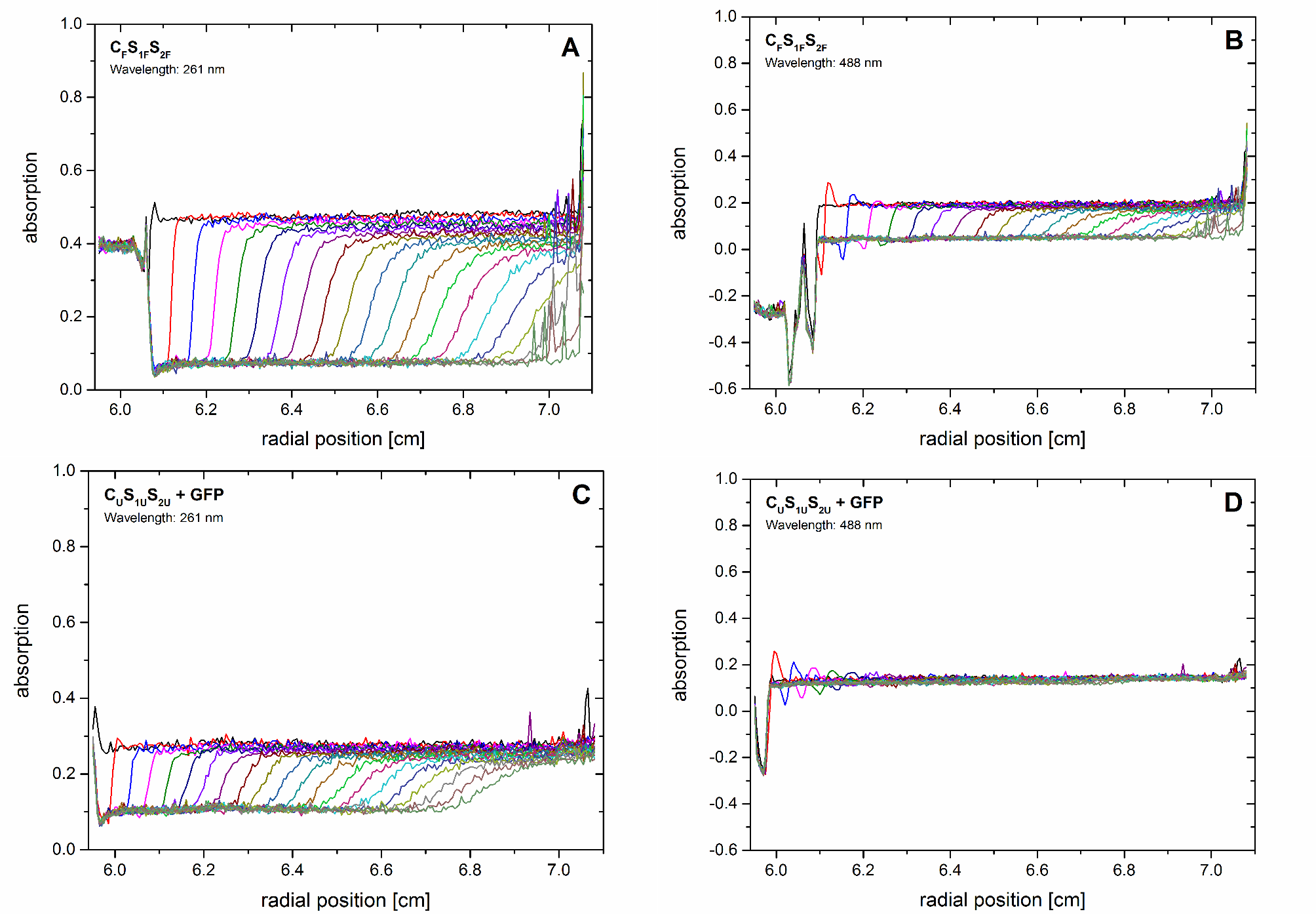

Supplement: Supplementary file 8 — AUC spectra of the GFP modified silica nanoparticles (CFS1FS2F) and unlabelled silica nanoparticles mixed with GFP (CUS1US2U). Same amounts of particles and GFP were used. In all spectra, the absorption was plotted against the radial position (cm). To detect the sedimentation velocity of silica nanoparticles, a wavelength of 261 nm was chosen, whereas a wavelength of 488 nm was used for the GFP detection. In A and B the sedimentation of GFP-labelled particles is shown and in C and D the sedimentation of unlabelled silica nanoparticles with additional added GFP. (TIFF 8201 kb) [file 11671_2017_2280_MOESM8_ESM.tif]
